# Nasopharyngeal SARS-CoV-2 viral loads in young children do not differ significantly from those in older children and adults

**DOI:** 10.1038/s41598-021-81934-w

**Published:** 2021-02-04

**Authors:** Sharline Madera, Emily Crawford, Charles Langelier, Nam K. Tran, Ed Thornborrow, Steve Miller, Joseph L. DeRisi

**Affiliations:** 1 Division of Infectious Diseases, Department of Medicine, University of California, San Francisco, CA USA; 2 Chan-Zuckerberg Biohub, San Francisco, CA USA; 3 Department of Microbiology and Immunology, University of California San Francisco, San Francisco, CA USA; 4 Department of Medicine and Biochemistry & Biophysics, University of California San Francisco, San Francisco, CA USA; 5 Department of Pathology and Laboratory Medicine, Davis School of Medicine, University of California, Sacramento, CA USA; 6 Department of Laboratory Medicine, University of California San Francisco, San Francisco, CA USA

**Keywords:** SARS-CoV-2, Viral epidemiology

## Abstract

The role of children in the spread of the SARS-CoV-2 coronavirus has become a matter of urgent debate as societies in the US and abroad consider how to safely reopen schools. Small studies have suggested higher viral loads in young children. Here we present a multicenter investigation on over five thousand SARS-CoV-2 cases confirmed by real-time reverse transcription (RT) PCR assay. Notably, we found no discernable difference in amount of viral nucleic acid among young children and adults.

## Introduction

Children remain underrepresented in current studies aimed to analyze the spread of SARS-CoV-2 coronavirus, making their contribution to viral transmission elusive. It is well established that, in general, children experience less severe illness than do adults, though in rare cases children can be subject to a severe multisystem inflammatory syndrome^1^. And there is an emerging view that children may play a lesser role in the spread of SARS-CoV-2 than they do in other respiratory pathogens^2^, but much uncertainty about this remains^3^. Recently, it was reported that children less than five years old may carry higher viral loads in the nasopharynx than older children and adults^4^, raising concerns that exposure to this group may pose special epidemiologic risks. Here we report results bearing on this question from two coronavirus testing laboratories that serve large populations of patients in California.

## Results

By providing widely available, free SARS-CoV-2 testing to the community, a total of 5,544 patients with laboratory-confirmed COVID-19 were identified. Laboratory A and B identified 4,619 and 925 patients. The population in Laboratory A was slightly younger than that of Laboratory B by mean and median (Table 1). Cases of COVID-19 were analyzed using three age categories, young children aged less than five years (n = 199), children aged five to 17 (n = 665), adults aged and older (n = 4680), were identified. Table 1 denotes baseline characteristics of laboratory-confirmed COVID-19 study participants by laboratory. Statistically significant differences between the population of Laboratory A and B include mean age, 36.5 and 42.4, respectively, as well as percentage of hospitalized cases, 4.4% and 24.4%, respectively.

**Table 1:** Characteristics of study population.

	Laboratory A	Laboratory B	p value
Total	4619	925	
Mean age (Years)	36.5	42.4	< .0001
Median age (Years)	35	41	
**Age (Years)**			
< 5	179	20	
5–17	617	48	
≥ 18	3823	857	
Male (%)	52.6%	51.5%	0.556
Female (%)	47.3%	48.4%	
Hospitalized (%)	4.4%	24.4%	< .0001
SymptomaGc (%)	NA	77.4%	

Baseline characteristics of study population for Laboratory A and B. *P* values calculated by two tailed t-test (Median Age) and Fischer’s Exact test (sex, hospitalization) are shown. *P* values less than 0.05 are considered significant.

**Figure 1:**
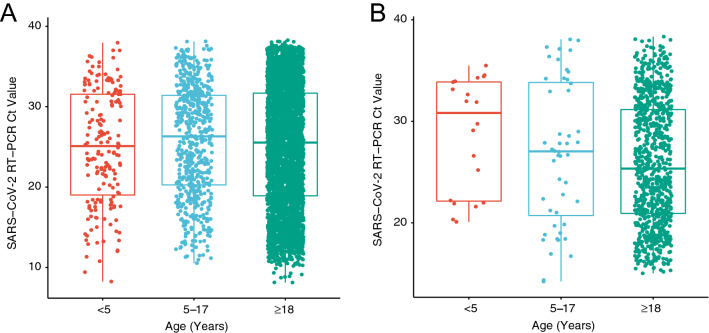
Age distributed nasopharyngeal SARS-CoV-2 viral nucleic acid content. SARS-CoV-2 viral nucleic acid detected by real-time RT-PCR in nasopharyngeal swabs from patients infected with SARS-CoV-2 as detected by (**A**) laboratory A (N = 4619, ANOVA *p* = 0.18) and (**B**) laboratory B (N = 925, ANOVA p = 0.073). Data are stratified by three age groups, ages < 5; 5–17; 18 and older.

Symptom status on testing was available at Laboratory B. Patients meeting Laboratory B symptom definition were defined as symptomatic (Supplemental Table 1A). Day of symptom onset for Laboratory B and symptom status for Laboratory A was unavailable. Age-specific stratification of hospitalization and symptom status by location can be found in Supplementary Table 1B. In order to ascertain potential differences in viral load, Ct values were assessed. As depicted in Fig. 1, despite differences in the population represented by Laboratory A and B, no significant differences in Ct value were observed across the three age groups. In particular, the children less than age 5 did not display higher nasopharyngeal viral loads than older children or adults. No differences across age groups were found when comparing viral loads for Laboratory A cases (Supplemental Fig. 1A), conversion of Ct values to viral loads were not available for Laboratory B cases.

Furthermore, Ct values did not differ significantly between hospitalized and non-hospitalized cases across both laboratories (Supplemental Fig. 1B and C). Comparison of Ct values across symptom status for Laboratory B cases yielded a statistically significant difference between young children aged less than five and adults of 18 years of age and older (Supplemental Fig. 1D). However, this difference is not considered clinically significant as the number of cases of asymptomatic young children was too low for appropriate comparison (age less than five n = 3, age 18 and older n = 193).

## Discussion

In contrast to a prior smaller study of 145 individuals^4^, our study of 5,544 children and adults did not demonstrate higher nasopharyngeal viral loads in children under five years of age. Notably, a significant proportion of Laboratory B outpatient cases were asymptomatic, underscoring the importance of broad community testing strategies. No clinically significant differences in Ct value or viral load were noted across age groups upon comparison of hospitalization status or symptom status. Differences in Ct values among asymptomatic children and adult cases in Laboratory B are not deemed clinically significant as there were only three asymptomatic children in that cohort. One limitation of this study is the lack of symptom status for outpatient cases of Laboratory A. A recent outpatient study supported by Laboratory A with a comparable demographic identified that 52% of patients were asymptomatic at the time of testing, which may provide a reasonable estimate for the cases originating from Laboratory A^5^. Our work largely presents outpatient cases and is relevant to the majority of pediatric COVID-19 cases but possibly not to the subset of children hospitalized with severe disease.

There are conflicting data on the association of viral load with disease severity, with some studies showing higher viral loads in severe cases^6^, while others indicate a lower viral load in hospitalized patients than those not hospitalized^7^. Since the viral load changes rapidly during early infection, the time between symptom onset and sampling is a significant variable. Some patients with severe disease may delay entry to care, missing the peak period of viral shedding. Notably, these earlier studies were done primarily in the hospital setting, so the findings do not necessarily translate to the outpatient population. While the presented work offers a large predominantly outpatient population, it is limited by an inability to compare time between sampling, symptom onset, and symptom severity, which remain significant variables. We believe this limitation does not take away from the overall conclusion due to the robust sample size of largely asymptomatic or mildly symptomatic individuals, making this work likely to be representative of the general population of infected subjects. Sampling bias can also compromise the generalizability of the obtained results, such as testing availability and cost to testing. However, testing was made widely available to the general population at no cost. Patients were allowed to self-present at will, or secondary to symptoms or due to contact tracing. Given the low barrier and wide availability to testing, we further regard these data to likely be applicable to the general population. An accurate understanding of the variables that affect viral transmission, including amount of virus carriage, will be essential to guide public policy efforts as re-opening strategies are devised.

We caution, however, that viral load as determined by RT-PCR is only one of many potential influences on infectivity. PCR accurately enumerates viral genomes, but does not indicate whether they come from infectious virions, defective viral particles, or lysed infected cells. Infectivity in populations is affected by many other clinical, behavioral and environmental factors. Our findings argue against the idea that young children are more infectious due to higher viral loads, and suggest an alternative explanation for their contribution to SARS-CoV-2 transmission, such as representing a reservoir of asymptomatic infections. Ultimately, future epidemiological studies are needed to understand the role of children in the spread of SARS-CoV-2.

## Methods

### Ethical considerations

All research was performed in accordance to UC San Francisco good clinical practice guidelines. The protocol was approved by UC San Francisco research committee. All data were anonymized and de-identified before analyses. The UC San Francisco Institutional Review Board provided an exemption and waiver of HIPAA authorization and informed consent.

### Sample collection and processing

Testing was carried out in two laboratories from March–August 2020. Laboratory A serves the University of California (UC) San Francisco health care system, local clinics and county health departments in 26 California counties. Laboratory B serves the UC Davis health care system and partner clinics/hospitals centered in Sacramento, CA. Testing was made available to the general population at no cost. Patients were encouraged to self-present for symptoms, as part of contact tracing, or at will. Nasopharyngeal swabs were collected at various outpatient, drive-through, inpatient, and emergency department testing sites. Laboratory A swabs were collected in DNA/RNA Shield (Zymo Research) to inactivate virus and preserve RNA stability. Laboratory B collected specimens into 3 mL Becton, Dickinson and Company (Franklin Laes, NJ)/Copan (Murrieta, CA) Universal Transport Medium (UTM) or Remel Viral Transport Medium (VTM) (San Diego, CA). Real RT-PCR used NEB Luna Universal RT-qPCR kit (New England Biolabs, Ipswitch MA) on Bio-Rad CFX384 instruments (Bio-Rad, Hercules, CA) in Laboratory A; and Roche cobas 6800 (Roche Diagnostics, Indianapolis, IN) using the EUA SARS-CoV-2 assay in Laboratory B. Each real-time RT–PCR assay provided a threshold cycle (Ct) value, indicating the number of cycles surpassing the threshold for a positive test. Samples were considered positive if the Ct value was ≤ 40, and otherwise it was negative. Viral loads for Laboratory A cases were converted using a viral load standard curve created on the establishment of CLIA-certified Laboratory A. Statistical significance was calculated using ANOVA, Student t-tests, or Fisher’s exact test. *P* values less than 0.05 were considered significant. Statistical analysis used R ggpubr v.0.4.0.

## Supplementary Information

Supplementary Figure 1.

Supplementary Table 1.

Supplementary Legends.

## Data Availability

The data shown in the manuscript are available upon request from the corresponding author.
