# Supplementary figures and images for: Nasopharyngeal SARS-CoV-2 viral loads in young children do not differ significantly from those in older children and adults
Source: Sci Rep. 2021 Feb 4;11:3044. doi: 10.1038/s41598-021-81934-w (PMC7862672; doi:10.1038/s41598-021-81934-w)

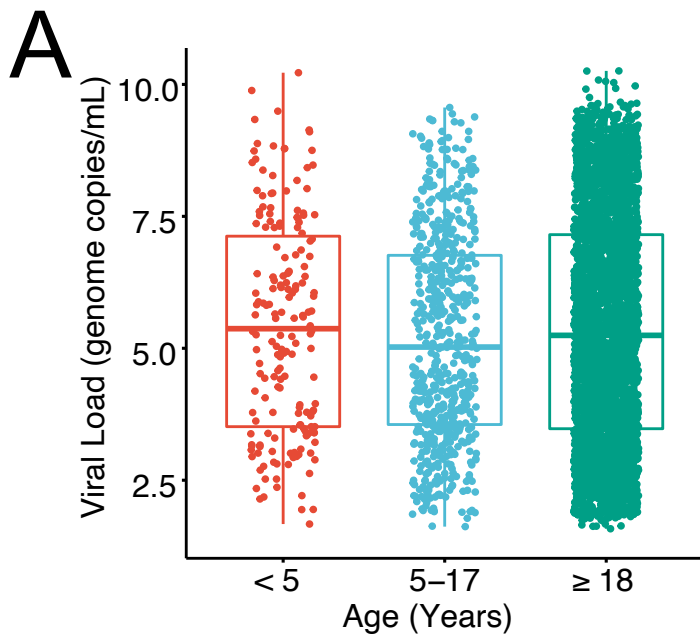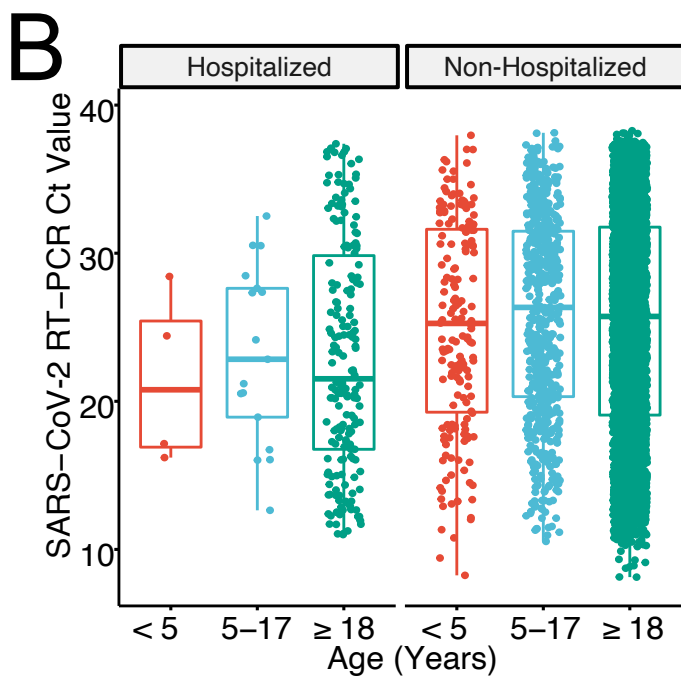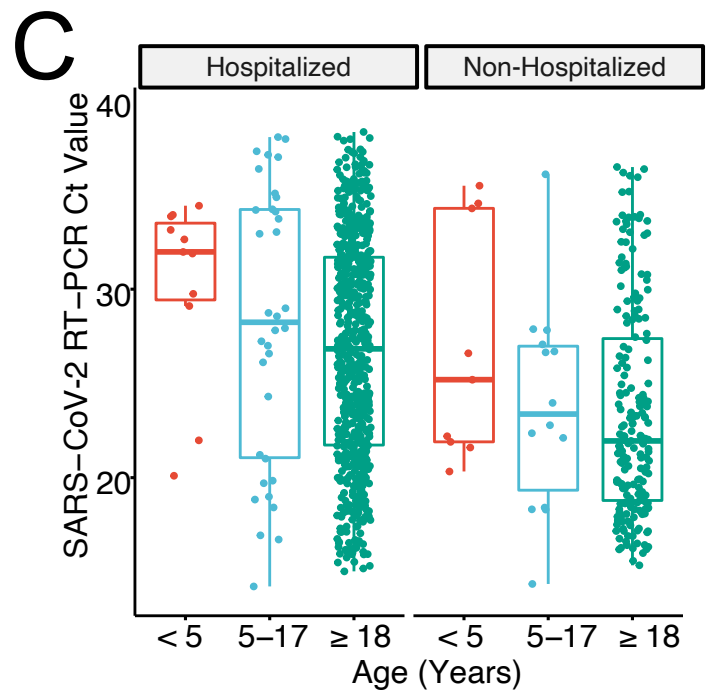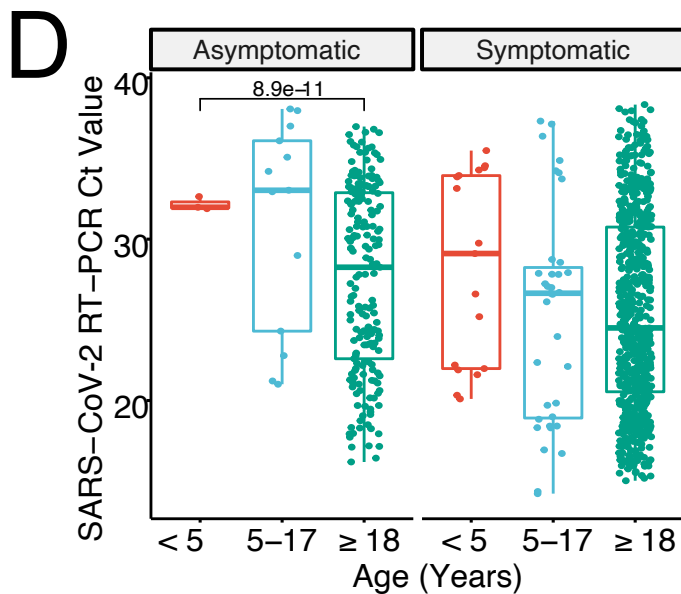

Supplement: Supplementary file 1 — Supplementary Figure 1. [file 41598_2021_81934_MOESM1_ESM.pdf]
