# Supplementary material for: Nasopharyngeal SARS-CoV-2 viral loads in young children do not differ significantly from those in older children and adults
Source: Sci Rep. 2021 Feb 4;11:3044. doi: 10.1038/s41598-021-81934-w (PMC7862672; doi:10.1038/s41598-021-81934-w)
Supplement: Supplementary file 2 — Supplementary Table 1. [file 41598_2021_81934_MOESM2_ESM.pdf]

A

Laboratory B Defined Symptoms

- Fever ≥ 100°F or 37.8°C
- Shortness of breath or difficulty breathing
- Chills
- Repeated shaking with chills
- Muscle pain
- Headache
- Sore throat (not due to a chronic condition)
- New loss of taste and smell
- Diarrhea

B

Laboratory A

Age (Years)	**<5**	**5-17**	**≥18**
Total	179	617	3823
Hospitalized	4	17	186
Non-hospitalized	175	600	3637

Laboratory B

Age (Years)	**<5**	**5-17**	**≥18**
Total	20	48	857
Hospitalized	9	14	203
Non-hospitalized	11	34	654
Symptomatic	17	35	664
Asymptomatic	3	13	193
