# Supplementary material for: Nasopharyngeal SARS-CoV-2 viral loads in young children do not differ significantly from those in older children and adults
Source: Sci Rep. 2021 Feb 4;11:3044. doi: 10.1038/s41598-021-81934-w (PMC7862672; doi:10.1038/s41598-021-81934-w)
Supplement: Supplementary file 3 — Supplementary Legends. [file 41598_2021_81934_MOESM3_ESM.docx]

Supplemental Table 1: Symptoms Definition and Laboratory-specific age stratification. Symptoms of SARS-CoV-2 infection defined by Laboratory B (a). Number of patients according to stratified age groups, ages <5; 5-17; 18 and older in Laboratory A and B (b).

Supplemental Figure 1: Age distributed nasopharyngeal SARS-CoV-2 burden. Viral loads converted from SARS-CoV-2 viral nucleic acid detected by real-time RT-PCR in nasopharyngeal swabs Ct values for Laboratory A (a). SARS-CoV-2 viral nucleic acid detected by real-time RT-PCR in nasopharyngeal swabs from hospitalized and non-hospitalized patients infected with SARS-CoV-2 as detected by (b) laboratory A (ANOVA hospitalized p = 0.93, non-hospitalized p= 0.23) and (c) laboratory B (ANOVA hospitalized p = 0.18, non-hospitalized p= 0.064); and symptomatic and asymptomatic patients infected with SARS-CoV-2 as detected by (b) laboratory B (ANOVA symptomatic p = 0.18, asymptomatic p= 0.047, further statistical analysis performed with Student’s t-test, p values ≤ 0.05 are depicted). Data are stratified by three age groups, ages <5; 5-17; 18 and older.
